# Specific Cytotoxic Effects of Parasporal Crystal Proteins Isolated from Native Saudi Arabian *Bacillus thuringiensis* Strains against Cervical Cancer Cells

**DOI:** 10.3390/molecules24030506

**Published:** 2019-01-31

**Authors:** Mourad A. M. Aboul-Soud, Mohammed Z. Al-Amri, Ashok Kumar, Yazeed A. Al-Sheikh, Abdelkader E. Ashour, Talat A. El-Kersh

**Affiliations:** 1 Chair of Medical and Molecular Genetics Research, Department of Clinical Laboratory Sciences, College of Applied Medical Sciences, King Saud University, P.O. Box 10219, Riyadh 11433, Saudi Arabia; mohd.zain2@hotmail.com (M.Z.A.-A.); yalsheikh@KSU.EDU.SA (Y.A.A.-S.); talatkersh@yahoo.com (T.A.E.-K.); 2 Cairo University Research Park, Cairo University, Giza 12613, Egypt; 3 Vitiligo Research Chair, College of Medicine, King Saud University, Riyadh 11433, P.O. Box 10219, Saudi Arabia; aknirankari@gmail.com; 4 Department of Pharmacology and Toxicology, College of Pharmacy, King Saud University, P.O. Box 2457, Riyadh 11451, Saudi Arabia; aeashour@gmail.com

**Keywords:** *Bacillus thuringiensis*, parasporin, δ-endotoxin, non-insecticidal inclusions, in vitro cytotoxicity, apoptosis

## Abstract

Currently, global efforts are being intensified towards the discovery of local *Bacillus thuringiensis* (Bt) isolates with unique anticancer properties. Parasporins (PS) are a group of Bt non-insecticidal crystal proteins with potential and specific in vitro anticancer activity. However, despite the significant therapeutic potential of PS-producing Bt strains, our current knowledge on the effects of these proteins is limited. Hence, the main objective of this study was to screen Bt-derived parasporal toxins for cytotoxic activities against colon (HT-29) and cervical (HeLa) cancerous cell lines. Nine non-larvicidal and non-hemolytic Bt strains, native to Saudi Arabia, were employed for the isolation of their parasporal toxins. *16S* rDNA sequencing revealed a 99.5% similarity with a reference Bt strain. While PCR screening results indicated the absence of selected *Cry* (*Cry4A*, *Cry4B*, *Cry10* and *Cry11*), *Cyt* (*Cyt1* and *Cyt2*) and *PS* (*PS2*, *PS3* and *PS4*) genes, it concluded presence of the *PS1* gene. SDS-PAGE analysis revealed that proteolytically-cleavaged PS protein profiles exhibit patterns resembling those observed with PS1Aa1, with major bands at 56 kDa and 17 kDa (Bt7), and 41 kDa and 16 kDa (Bt5). Solubilized and trypsinized PS proteins from all Bt strains exhibited a marked and dose-dependent cytotoxicity against HeLa cancerous cells but not against HT-29 cells. IC_50_ values ranged from 3.2 (Bt1) to 14.2 (Bt6) with an average of 6.8 µg/mL. The observed cytotoxicity of PS proteins against HeLa cells was specific as it was not evident against normal uterus smooth muscle cells. RT-qPCR analysis revealed the overexpression of *caspase 3* and *caspase 9* by 3.7, and 4.2 folds, respectively, indicative of the engagement of intrinsic pathway of apoptosis. To the best of our knowledge, this is the first report exploring and exploiting the versatile repertoire of Saudi Arabian environmental niches for the isolation of native and possibly novel Saudi Bt strains with unique and specific anticancer activity. In conclusion, native Saudi Bt-derived PS proteins might have a potential to join the arsenal of natural anticancer drugs.

## 1. Introduction

Cancer is one of the leading causes of death worldwide and therefore it poses a major global public health concern. In 2018, a striking rise in global cancer burden has been reported with 18.1 million newly diagnosed cases, 9.6 million mortalities and 43.8 million people 5-year survival rate compared to only 12.4, 7.6 and 28 million people in 2008, respectively. In 2030, it is projected that these figures will continue to rise to an alarming 26.4 million newly diagnosed cases and 17 million cancer-related deaths [1]. Worldwide, lung, breast and colorectal cancers account for one third of cancer incidence and mortality burden, are classified in the top three highly prevalent cancer types, and ranked within the top five in terms of death rates (1st, 5th and 2nd, respectively). According to gender, colorectal cancer (CRC) ranks third (10.9%) in terms of incidence in males, whereas breast cancer (BC) ranks first in women (24.2%). Cervical cancer ranks fourth for both. Cervical cancer leads the race for both incidence (6.6%) and mortality (7.5%) being ranked fourth for both in women [1]. In Saudi Arabia, cancer prevalence is also affected by gender, with CRC (13.3%) and BC (25.8%) are the most predominant types in men and women, respectively [2]. By the year 2030, it has been predicted that the CRC incidence in Saudi Arabia would increase four-fold in both genders. Moreover, it is estimated that by year 2025, incidence and mortality rates will significantly rise by 350% and 160%, respectively [3]. Therefore, countries should be prepared to face the challenge of a foreseeable increase in cancer burden. This is mainly attributed to the population growth, adoption of modern lifestyles and its associated risk factors (e.g., tobacco smoking, increased consumption of meat and adopting sedentary way of life that lead to an increase in the body mass index and obesity) and aging of the population [4]. Despite the fact that conventional anticancer therapies (i.e., surgical resection, radiotherapy and chemotherapy) are effective in the management of patients, they are however ineffective in approximately 50% of malignancy cases. Unfortunately, currently employed synthetic cytotoxic drugs can be associated with a range of side effects including neutropenia, oral ulceration, diarrhea, hair loss, and nerve and kidney damage [5]. Therefore, substantial efforts are currently being focused on the development of novel and natural alternative therapeutic techniques for the efficient targeting of tumors. 

*Bacillus thuringiensis* (Bt) is a Gram-positive spore-forming bacterium that is uniquely characterized by the production of proteinaceous crystalline parasporal inclusions in sporulating cells. Insecticidal Bt produces parasporal inclusions (proteins) collectively known as δ-endotoxin. These parasporal inclusions are encoded by a family of *Cry* and *Cyt* genes [6]. For decades, these Bt parasporal proteins have been successfully employed as biological control agents [7] and they are currently classified and named based on their protein identities according to norms provided by the Bt δ-endotoxin nomenclature committee [8,9], whereby ensuring that closely related toxins are ranked together. The economic importance and long use in biological pest management programs of Cry proteins has stirred a significant amount of work primarily focused on the elucidation of their mode-of-action [10]. Therefore, the mode-of-action of δ-endotoxin proteins is thought to involve: (i) solubilization of the crystal in the insect′s midgut, (ii) proteolytic activation of the protoxin by midgut proteases, (iii) binding of the Cry toxin to midgut glycoprotein receptors, which are located at the surface of the apical brush border membrane of epithelial columnar cells, and (iv) insertion of the toxin into the apical membrane, where it creates pores (ion channels) and eventually kills susceptible insects [11]. 

Research reports from our lab and from others concluded that Bt isolates with non-insecticidal Cry proteins are more prevalently distributed than insecticidal ones in natural environmental niches, accounting for more than 90% of the natural populations from soils and phylloplanes [12,13]. This observation has stimulated a new line of research questioning whether the non-insecticidal Cry proteins possess some as yet unidentified novel biological functions. These efforts have subsequently revealed that Cry proteins produced by non-insecticidal Bt isolates exhibit unique cytocidal activities against human cancer cells [14]. This anticancer activity has been attributed to the presence of a new family of Bt parasporal inclusions designated as parasporin (PS) [15]. PS has been defined as “*Bacillus thuringiensis* and related bacterial parasporal proteins that are non-hemolytic but capable of preferentially killing cancerous cells” [16]. However, it has to be noted that Cry and PS terms are not mutually exclusive as some PS proteins may possess both insecticidal and cancer cytocidal activities. PS was first identified as a human leukemic cell-recognizing parasporal protein [15]. Similar to Cry toxins, PS molecules are synthesized as nascent protoxins that require proteoloytic processing to exert its full cytotoxic potential [13]. However, despite the structural resemblance of PS proteins with Cry toxins, they only exhibit less than 25% of amino acid sequence homology with them, thus they require a distinct classification system. Accordingly, the Committee of Parasporin Classification and Nomenclature (http://parasporin.fitc.pref.fukuoka.jp/) has been established to construct a taxonomically-sound quaternary rank nomenclature systems based on the amino acid identity, similar to that followed with Cry toxins [17]. Initially, there were six first-rank categories of PS (PS1 to PS4) [17], but soon this was updated to include two new subgroups (PS5 and PS6) [18,19]. Currently immense research efforts are being devoted to the discovery of novel classes of PS proteins. The abundance of these bacilli in nature and their selectivity have made them potential candidates for cancer treatment. However, literature on the in vivo effect of these proteins is scarce. Since different Bt strains produce different cytotoxic proteins with wide variations in their anticancer effect and mechanism-of-action, further investigations are necessary and their effect in vivo must be well established before they can be used in clinical trials. Many countries including Saudi Arabia (our research team) are continuing to screen and isolate novel strains in natural environments to apply for biological pest management programs whereby reducing insect resistance to Bt [12,20,21,22,23]. Moreover, global efforts are currently being intensified towards the discovery of local Bt isolates with unique anticancer properties [13,19,24,25,26]. 

In the light of the potential application of these ubiquitous proteins in cancer therapy, this study was undertaken to characterize nine new non-larvicidal and non-hemolytic Bt isolates collected from various regions in Saudi Arabia according to: (i) colony and crystal morphology, (ii) protein profile of the crystalline parasporal inclusions, (iii) screening for presence of *Cry*, *Cyt* and *PS* genes by PCR, (iv) screening for in vitro cytotoxic activities against two selected cancerous cell lines; viz., colon (HT-29) and cervical (HeLa), and (v) mechanism of killing.

## 2. Results

### 2.1. Phase Contrast and Electron Microscopy

**Figure 1:**
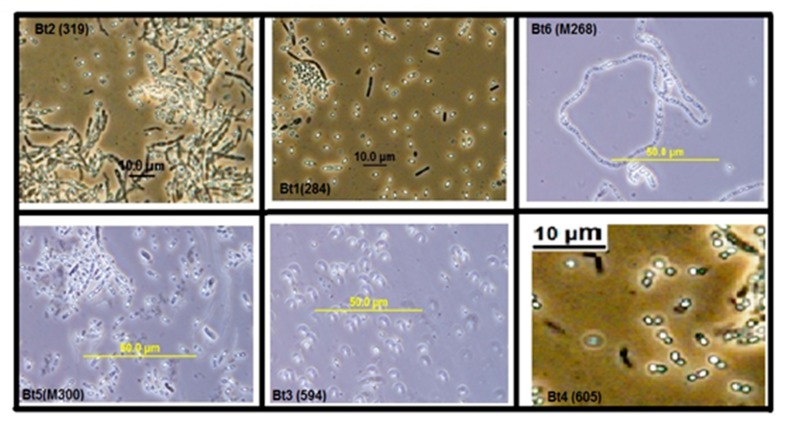
Phase contrast micrographs of some native Bt isolates. Morphology of parasporal crystals from Bt1, Bt2, Bt3, Bt4, Bt5, Bt6 is bi-pyramidal, spherical, small bi-pyramidal, attached crystals, ovoid and hexagonal, respectively. Within ’parentheses’ are their respective original identification numbers.

**Figure 2:**
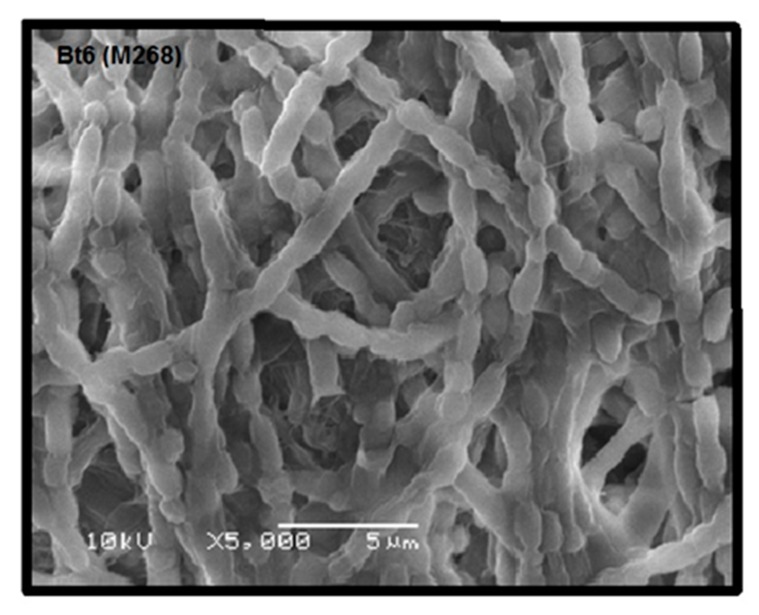
Scanning electron micrograph of Bt6 isolate at magnification of 5000× showing characteristic hexagonal attached crystals (arrow head).

Colonies of the recovered nine Bt isolates on nutrient agar supplemented (NAS) medium appeared as circular, scalloped-edged, non-pigment, off-white with irregular margins and slightly centered (data not shown). Colonies were tentatively classified into three groups (A to C). A: off-white, round, mucoid, slightly raised center; B: off-white, shiny, little-raised center, and irregular margin, and C: off-white, round, slightly raised center. The ratios for colonial morphology of the Bt strains were type A = 55.56%, B = 33.33% and C = 11.11%. Phase contrast microscopy revealed that all Bt strains shared many morphological characteristics in cell chains arrangement, ellipsoidal shape of spores, and sporangia, in agreement with our previously published data [27]. The nine Bt isolates under investigation were classified into four classes according to diverse morphological characteristics of its parasporal crystals, viz., spherical, hexagonal, bi-pyramidal and ovoid (Figure 1). It is worth noting that spherical and hexagonal crystals were the most predominant types among the studied Bt isolates, conforming to our recently published report [28]. To confirm the above observed crystal shape of selected Bt isolates, some isolates were also examined by scanning electron microscopy (SEM). For example, Bt6 (M268) examination under SEM clearly revealed the typical hexagonal attached crystal parasporal inclusions with better resolution and clarity (Figure 2). 

### 2.2. Biochemical and 16S Molecular Typing of Isolated Bt Strains

All Bt isolates were negative for H_2_S = Sodium thiosulfate, URE = Urea, IND = l-tryptophan, SOR = d-sorbitol, RHA = l-rhamnose and MEL = d-melibiose. For SAC (Sucrose) there were three positives (Bt1, 4 and 5) and the other six were negative. For AMY (starch hydrolysis), all strains were positive except Bt6 and 8. For VP (Sodium pyruvate), six isolates (Bt1, 2, 5, 6, 8 and 9) were positive, whereas the other three were negative. For CIT (Citrate), there were six positives, with the exception for Bt4, 6 and 8. As for OX (Oxidase), all were positive except Bt3. With respect to MOT (Motility), four were motile (Bt1, 3, 4 and 5) while the other four were non-motile. All strains were positive for arginine dihydrolase (ADH) activity with the exception of Bt3, 6 and 8 (Table 1). As for 16S typing, target amplicon bands (~1550 bp) were detected by agarose gel electrophoresis, for all tested Bt strains and reference strains (*Bti*-H14 and *B. cereus*) before being sequenced (data not shown). Phylogenetic data analysis of the obtained *16S rDNA* gene sequences indicated that the nine tested isolates shared a high degree of homology (up to 99.5%) to *Bti*-H14 reference strain, which is in agreement with our previously published data [28]. 

### 2.3. PCR Analysis of Bt Isolates

**Figure 3:**
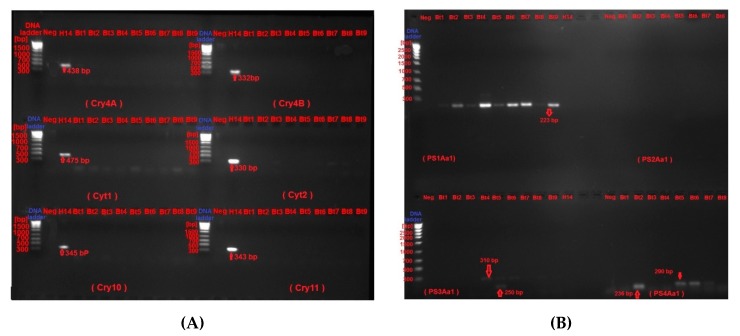
Polymerase chain reaction screening of Bt isolates for a panel of *Cry*, *Cyt* and *PS* genes. PCR reactions were conducted as described in Materials and Methods section. Amplicons were run at 100 v for 30 min in 2% agarose gels pre-stained with ethidium bromide and examined with a charge-coupled device (CCD) camera mounted on a UVITC gel documentation system. Standard DNA marker was included in lane 1 to identify band sizes. (**A**) PCR of four *Cry* (4A, 4B, 10 and 11) *Cyt* (1 and 2) genes. All nine Bt strains were negative for these genes except the reference strain *Bacillus thuringiensis subsp. israelensis* (*Bti-*H14), which were positive for all tested *Cry* and *Cty* genes. (**B**) PCR for parasporin genes *PS1*, *PS2*, *PS3* and *PS4*. All nine strains were positive for PS1 but negative for the rest. Neg., denotes negative control lane where deionized water was included instead of plasmid DNA template.

Polymerase chain reaction (PCR) was performed on plasmid DNA to screen for the presence of selected *Cry*, *Cyt* and *PS* genes. Results of agarose gel electrophoresis revealed that were negative for all tested *Cry* and *Cyt* genes when compared to the reference strain *Bti-*H14, which was positive for all of them (*Cry4A*, *Cry4B*, *Cry10*, *Cry11*, *Cyt1* and *Cyt2*). On the other hand, while the nine tested strains were negative for *PS2*, *PS3* and *PS4* genes, they exhibited positive amplification for target amplicon of the *PS1* gene. As expected, the reference strain *Bti-*H14 exhibited no amplifications of the target products for all tested *PS* genes (Figure 3).

### 2.4. SDS-PAGE Analysis of Nascent and Trypsin-Activated PS Proteins

**Figure 4:**
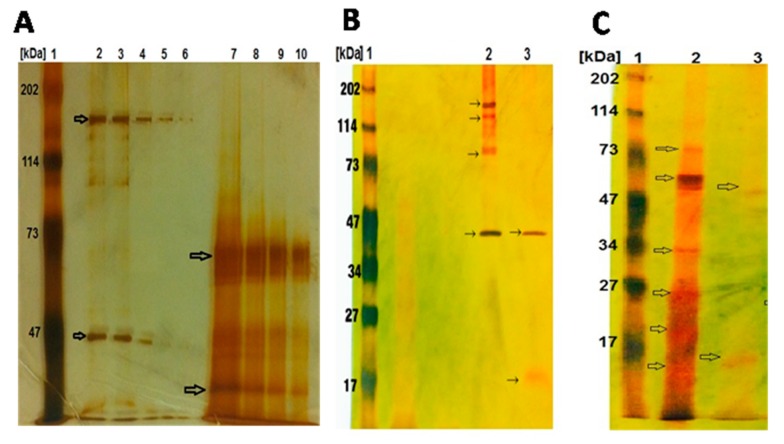
SDS-PAGE analysis of solubilized native and activated Bt parasporal crystal inclusions. PS proteins was resolved onto 12% SDS-PAGE gel and the bands were detected by silver staining, which was performed as detailed in the methods section. (**A**) Protein profiles of nascent PS proteins from Bt5 and Bt7. Lanes 2–6 were loaded with PS proteins from Bt5 at 10 µg (2&3), 3.6 µg (4), 1.6 µg (5) and 0.4 µg (6); major bands at 155 kDa and 41 kDa, and light bands at 140 kDa, 114 kDa and 105 kDa. PS proteins from Bt7 were loaded into lanes 7–10 at 10 µg (7), 3.6 µg (8), 1.6 µg (9) and 0.4 µg (10); major bands at 68 kDa, 62 kDa and 30 kDa, light bands at 43 kDa, 39 kDa and 37 kDa. (**B**) Protein profiles of nascent and activated PS proteins from Bt5. Lane 1, molecular marker; lane 2, loaded with 10 µg native un-activated PS proteins with major bands at 155 kDa, 140 kDa, 82 kDa and 41 kDa; lane 3, trypsinized PS proteins with major bands at 41 kDa and 16 kDa. A trypsin-resistant band at molecular size of 41 kDa is evident. (**C**) Protein profiles of nascent and activated PS proteins from Bt7. Lane 2, loaded with 9 µg native un-activated PS proteins with major band at 68 kDa, and light bands at 83 kDa, 64 kDa, 32 kDa, 25 kDa, 19 kDa and 15 kDa; lane 3, loaded with 9 µg trypsinized PS proteins with light bands at 56 kDa and 16 kDa.

SDS-PAGE of solubilized native and activated Bt parasporal crystal inclusions was conducted in order to examine PS banding pattern before and after activation (Figure 4). Silver-stained PS proteins that had been resolved onto 12% SDS-PAGE gel exhibited strain-specific protein banding profiles. For example, profile of nascent PS proteins from Bt5 showed bands at 155 kDa, 140 kDa, 82 kDa and 41 kDa, whereas PS proteins from Bt7 showed bands at 83k Da, 68 kDa, 64 kDa, 32 kDa, 25 kDa, 19 kDa and 15 kDa (Figure 4A). Upon trypsin activation, while PS proteins from Bt5 showed two bands at 41 kDa and 16 kDa (Figure 4B), PS proteins from Bt7 showed two bands at 56 kDa and 16 kDa (Figure 4C). Strains Bt1, 2, 3 and 4 exhibited identical protein profiles to Bt 5, whereas Bt8 and 9 exhibited profiles identical to Bt7.

### 2.5. Specific Anti-Proliferative Effects of PS Proteins

Cytocidal activities of solubilized and trypsin-digested parasporal inclusion crystal proteins were assayed against cervical and colon cancer cells, viz., HeLa and HT-29, respectively, as judged by the observed cytopathic effect under phase contrast microscopy (data not shown). To this end, 3-[4,5-dimethylthiazol-2-yl]-2,5-diphenyltetrazoliumbromide (MTT) cytotoxicity data revealed differential and dose-dependent anti-proliferative effect only against HeLa but not HT-29 cells (Table 2). In this context, alkali-solubilized and trypsinized PS proteins at a concentration of 12.5 µg/mL, which had been recovered from the nine Bt strains under investigation, resulted in significant proliferation inhibition rates against HeLa cells that ranged from 38 to 47%, averaging 40%, whereas, IC_50_ values ranged from 3.2 (Bt1) to 14.2 (Bt6) averaging 6.8 µg/mL. The anticancer drug dasatinib exhibited inhibition and IC_50_ values of 58% and 24.4 µg/mL, respectively. By contrast, HeLa cells exhibited minimal susceptibility against alkali-solubilized PS proteins in the absence trypsinization and upon heat denaturation (94 °C for 5 min) of solubilized and proteolyzed PS proteins. The observed cytotoxicity of PS proteins against HeLa cells was specific as it was not evident against normal uterus smooth muscle cells (UtSMC). 

### 2.6. Transcriptional Activation of Apoptosis-Related Genes

**Figure 5:**
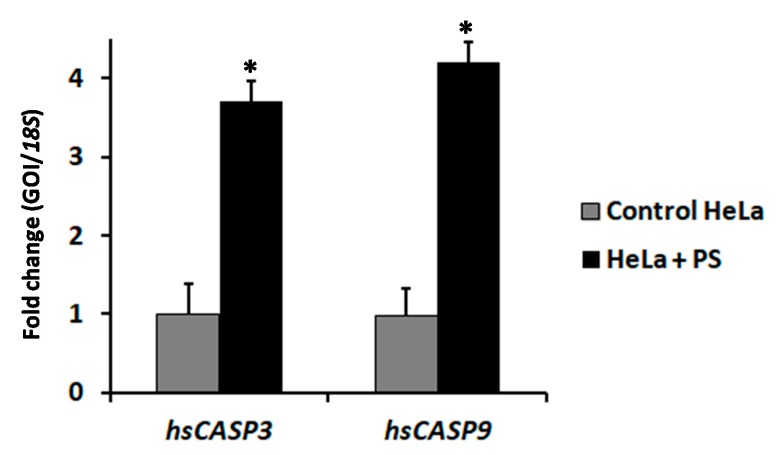
RT-qPCR analysis of apoptosis-related genes in HeLa cells. HeLa cells were treated for 24 h with 12.5 µg/mL of solubilized and activated PS proteins from Bt5. Expression profile was comparable with that obtained with Bt7. Relative expression was determined with the 2^−ΔΔCt^ method using the PCR efficiencies determined with the standard curve included in each run. Each data point represents the results obtained from three independent batches of cDNA made. Expression of target genes (Gene of interest (GOI), *hsCASP3* and *hsCASP9*) was normalized to that of the housekeeping gene *18S*, represented as mean ± SD, and is expressed as fold-change. (*) indicates significantly different mean values at *p* < 0.05 using a paired Student’s *t*-test.

The effect of alkali-solubilized and proteolytically-activated PS proteins (12.5 µg) from Bt5 on the transcriptional activity of two key apoptosis-related genes (i.e., caspase 3, *CASP3*; and caspase 9, *CASP9*) in HeLa cells was investigated by quantitative reverse transcription PCR (RT-qPCR) 24-h post-treatment (Figure 5). Results of RT-qPCR revealed a significant (*p* < 0.05) up-regulation in the level of expression for the two genes examined, in response to PS proteins, compared with Na_2_CO_3_ buffer control of identical dilution. Fold-change values of 3.7 ± 0.26 and 4.2 ± 0.27 were recorded for *hsCASP3* and *hsCASP9*, respectively, as compared with the control.

## 3. Discussion

Cancers are among the most severe threats to human health due to the unsatisfactory efficacy and inevitable systemic toxicity of traditional radio- and chemo-therapies. Recently, several experimental cancer treatments have been presented as medical therapies intended to treat cancer by improving, supplementing or replacing conventional methods. These include photodynamic therapy, HAMLET (human alphalactalbumin made lethal to tumor cells) [29], gene therapy [30], telomerase inhibition therapy [31], Photothermal therapy via femtosecond-laser-excited nanoparticles [32], dichloroacetate (DCA) [33], non-invasive Kanzius radio frequency (RF) cancer treatment [34], complementary and alternative medicine (CAM) [35], nutritional therapy [36] and bacterial treatment [37]. But many of these therapies are controversial due to lack of efficacy, specificity and selectivity. In spite of the known limitations of these therapeutic strategies, cancer can be cured provided that it is diagnosed at early stages. In the quest for finding an alternative cure for cancer that is more effective and specific, our research group has collected a repository of 68 Bt strains from 300 diverse environmental samples (October 2013–March 2014), isolated out of 16 sites across Saudi Arabia, and their potential larvicidal activity has previously been fully explored [12,27,38]. The present work represents the first report that examines the anticancer activity of nine non-larvicidal and non-hemolytic native *Bacillus thuringiensis* (Bt) isolates from different regions across Saudi Arabia.

The nine Bt strains were microscopically examined by both light and scanning electron microscopy, which confirmed the presence of the parasporal crystals of diverse morphologies, which conforms to previous findings [39]. Recently, it has been reported that PS proteins with non-insecticidal properties similarly exhibit variations in crystal morphology; which varies from spherical, bi-pyramidal to irregular [40]. The observed variation in crystal morphology correlate with the diversity of crystal proteins types in Bt isolates [9,41]. Moreover, the strains under investigation were molecularly confirmed to be *Bacillus thuringienesis* based on 99.5% homology to the reference strain *Bti*-H14, which is supported by our previous findings [28]. Analysis of PCR screening results indicated the absence of all the *Cry* and *Cyt* genes tested. This could be explained by the fact that all tested Bt isolates had previously been confirmed to be non-larvicidal and non-hemolytic; the two main characteristics generally found in Bt strains harboring PS proteins with anticancer activities [15,16]; which does not exclude the possibility that some PS proteins may be exhibiting insecticidal as well as anticancer activities [42]. The absence of Cyt proteins may explain the non-hemolytic nature of the tested Bt strains [7,8]. Moreover, PCR data concluded that Bt isolates harbor only the *PS1* gene but not *PS2*, *PS3* or *PS4*. Next, we attempted to group Bt isolates according to PS crystal protein(s) profile examined via SDS-PAGE. All the tested Bt isolates exhibited distinct nascent PS protein profiles from each other suggesting that the PS proteins could be different. Upon proteolytic cleavage, PS protein profiles exhibit patterns resembling those observed with PS1Aa1, with major bands at 56 kDa and 17 kDa (Bt7), and 41 kDa and 16 kDa (Bt5). PS1Aa1 protein isolated from strain Bt A1190 has been reported to have a molecular weight of 81 kDa for the nascent form, which is cleaved into two forms comprising 56 kDa and 15 kDa polypeptides upon activation [16]. The observed variation between the activated PS protein profiles of our Bt strains and the standard PS1Aa1 might point to some novelty, which remains to be verified by PCR amplicon sequencing and N-terminal sequencing. All of our tested alkali-solubilized and trypsinized PS proteins from Bt strains exhibited potent and specific cytotoxicity against HeLa cell lines, which is in agreement with previous report implicating potency spectrum of activated PS1Aa1 not only against cervical cancer but also leukemic and hepatocarcinoma cells [16]. This observation clearly indicates a heterogeneous nature in the spectrum of cytotoxicity conveyed by PS1 proteins. PS1Aa1 is structurally categorized into the three-domain type of PS proteins that resembles three-domain Cry toxins in possessing highly conserved five-block sequences [43]; their nascent polypeptides are about 80 kDa that are processed into 60–70 kDa active forms. The three-domain type of PS proteins is not only confined to PS1 [16] but also extends to include PS3 [44] and PS6 [19]. 

The mechanism-of-action of PS1Aa1 in susceptible cells, such as HeLa, has been proposed to involve a rapid stimulation of calcium influx leading to a concomitant increase in intracellular calcium concentration culminating in the engagement of apoptotic signaling transduction pathway [45]. To this end, we have profiled the expression levels of two apoptosis-related marker genes, viz., capsase 3 (*hsCASP3*) and caspase 9 (*hsCASP9*) by RT-qPCR. The significant up-regulation in mRNA expression levels of *hsCASP3* and *hsCASP9* genes supports the hypothesis that the observed susceptibility of HeLa cells towards PS proteins is mediated by activating the extrinsic apoptosis pathway. The intrinsic pathway is believed to be engaged at the cell surface via death receptor-mediated activation of either CASP9, or CASP8/CASP10 as the main initiators, leading to the activation CASP3 that is considered as an effector. It is noteworthy that once CASP3 is activated the apoptotic signaling cascade cannot be reversed or stopped [46].

## 4. Materials and Methods

### 4.1. Ethical Approval

The study was ethically approved by the Institutional Review Board of the College of Applied Medical Sciences, King Saud University (CAMS29/3334) on May 27th, 2013.

### 4.2. Chemical and Supplies

MTT (3-[4,5-dimethylthiazol-2yl]-2.5-diphenylterazolium bromide) was purchased from Sigma Aldrich (St Louis, MO, USA). DMEM, FBS, l-glutamine and penicillin/streptomycin were obtained from Hyclone Laboratories (Logan, UT, USA). Primers for 16s sequencing, PCR and RT-qPCR were ordered from Macrogen (Seoul, South Korea). Kits for cDNA reverse transcription and SYBR Green master mix were purchased from Qiagen (Hilden, Germany). All protein chemistry reagents and buffers were obtained from Bio-Rad Laboratories GmbH (Munich, Germany). 

### 4.3. Isolation, Culturing and Identification of B. Thuringiensis Isolates

A total of sixty-eight *B. thuringiensis* (BT) isolates were obtained (October 2013–March 2014) from diverse environmental samples recovered from various locations in Saudi Arabia according to previously published protocols by our group. The full procedures of isolation, culturing and identification of Bt strains are detailed therein [12,27,38]. 

### 4.4. Biochemical and Molecular Typing

Bt isolates were biochemically typed by use of API 20E systems (BioMerieux, Marcyle Etoile, France) according to the manufacturer′s instructions. Readings were carried out in duplicate after 24 h and 48 h of incubation. The isolates were classified according to biochemical types based on hydrolysis of SAC, sucrose and AMY, starch hydrolysis; VP, acetoin formation; CTA, citrate utilization; OX, oxidase; MOT, motility; ADH, arginine dihydrolase; and urea hydrolysis. *16S* rDNA gene sequencing was conducted to confirm the genotype of the isolated strains as compared to the reference strains *Bacillus thuringiensis* subsp. *israelensis* (*Bti-*H14) and *B. cereus* (ATCC1177). Briefly, Bt isolates were sub-cultured on Luria Bertani (LB) agar and incubated at 30 °C overnight. After incubation, a single colony was suspended into 100 µL sterile SDW water in an Eppendorf tube and placed in a shaking boiling water bath for 10 min followed by an immediate cooling shock at −20 °C. This heat-shock process was repeated three times to allow complete cell-lysis before centrifugation (Multifuge^TM^ 3SR Plus centrifuge, Thermo Scientific, Germany) at 10,000 rpm for 10 min at 4 °C. Template gDNA (1 µL) was added in a 20 µL PCR reaction comprising universal primers 27F(5′-AGAGTTTGATCCTGGCTCAG-3′/1492R(5′-TACGGTTACCTTGTTACGACTT-3′), and then a program of 35 amplification cycles (94 °C for 45 s, 55 °C for 60 s, and 72 °C for 60 s) was performed. Amplicons of ~1400 bp were obtained and subsequently purified to remove unincorporated PCR primers and dNTPs from PCR products by use of Montage PCR Cleanup Kit (Millipore, Burlington, MA, USA). Sequencing was performed by using Big Dye terminator cycle sequencing Kit (Applied Biosystems, Foster City, CA, USA) based on Sanger dideoxy chain termination method [47]. Sequencing products were resolved on an Applied Biosystems model 3730XL automated DNA sequencing system (Applied Biosystems, USA). The obtained sequences were manually cleaned and edited by use of BioEdit Sequence Alignment Editor [48]. Database search for sequences of annotated genes corresponding to obtained rDNA sequences was carried out using the NCBI nucleotide Blast (http://blast.ncbi.nlm.nih.gov). 

### 4.5. Scanning Electron Microscopy

In order to obtain the spore-crystal mixture, Bt isolates were grown in nutrient agar medium for 5 days at 30 °C, until lysis. The spore-crystal mixtures were suspended in 1 mL of ice-cold 1 M NaCl and centrifuged for 5 min at 13,000× *g* then washed 3 times with cold sterile distilled water. The pellets were suspended in distilled water. Diluted suspensions of spore-crystal complexes were placed on cover glasses and air dried. Sample were then examined and photographed with a FEI-Inspect S50, scanning electron microscope operating at a voltage of 1500 kV with 24,000× magnification [41].

### 4.6. Cell Lines and Culture Conditions

Two cancerous cell lines were utilized in this study, namely human cervical cancer (HeLa) and human colon adenocarcinoma (HT-29), were routinely maintained in DMEM (Dulbecco’s modified Eagle medium) supplemented with 10% FBS (Fetal Bovine Serum) and 2 mM l-glutamine and 1% penicillin/streptomycin. The cells were kept in sterile Nunc^TM^ cell culture treated polystyrene 75 cm^2^ flasks with vented filter cap (ThermoFisher Scientific™ GmbH, Karlsruhe, Germany), and sub-confluent cultures (70–80%) were trypsinized (Trypsin 0.05%/0.53 mM EDTA) and spilt depending on the seeding ratio. The cells were grown in a standard cell culture incubator at 37 °C in 5% CO_2_ humidified air.

### 4.7. Hemolytic Assay

Hemolytic activity of isolated PS proteins was determined against human erythrocytes (RBCs) according to standard procedures with some modifications [49]. Briefly, RBCs were washed three times in PBS and its concentration was adjusted to to 1 × 10^6^ cells/mL. Then, 100 µg of PS proteins was added per 1 mL RBCs and incubated for 18 h at 37 °C followed by centrifugation at 1500× *g* for 10 min. Finally, hemoglobin content in the resultant supernatant was measured at 570 nm by use of Shimadzu UV-1280 UV-VIS spectrophotometer (Nakagyo-ku, Kyoto, Japan).

### 4.8. Crystal Sporal Mixture Isolation

Bt isolates were inoculated to Modified Glucose Media (MGM) broth in separate flasks and incubated for 72 h. The pH of the culture broth of each isolate was brought down to 7 using 1 N HCl and centrifuged at 8000 rpm for 20 min. The supernatant was discarded and the pellet was resuspended in 6% lactose at one-tenth volume of initial broth. The suspension was stirred for 30 min and four-volume acetone was slowly added followed by stirring for another 30 min. Then, it was allowed to stand for 10 min at room temperature and filtered through Whatman No.1 filter paper. The acetone washing step was repeated thrice and finally the residue on the filter paper was allowed to dry overnight in a vacuum desiccator at room temperature. The white crystalline pellets were collected after drying and stored at 4 °C for further use [50].

### 4.9. Solubilization and Activation of the Crude Parasporal Crystal Proteins

The acetone powder containing crude PS proteins was suspended in 10 mL sterile deionized water (SDW) followed by centrifugation under cooling at 4000 rpm for 20 min. The pellet was then suspended in 3 mL SDW and washed twice with cold SDW via centrifugation. The washed pellet was then dissolved in 50 mM Na_2_CO_3_/HCl (pH 10.5), containing 10 mM dithiothreitol (DTT), EDTA (1 mM) for 3 h in a shaking incubator at 37 °C, followed by centrifugation at 4000 rpm for 50 min at 4 °C. The resultant supernatant was subjected to pH adjustment to 8 with 1 N HCl, followed by protein determination [51] and stored at −20 °C until further use. For proteolytic activation, crude proteins (1 mg/mL) was treated with trypsin at a final concentration of 0.2 mg/mL for 3 h at 37 °C. This was followed by further centrifugation at 20,000× *g* for 5 min to remove the remaining insoluble materials. Phenylmethylsulfonyl fluoride (PMSF, Wako Pure Chemicals, Japan) was added to the solution at a final concentration of 1 mM to stop the proteolytic reaction. The activated samples were sterilized by passing through 0.45 µm sterile syringe filter (Nalgene, Thermo Scientific) and utilized for MTT cytotoxicity and protein assays.

### 4.10. PCR Screening of Bt Isolates for Parasporin (PS) Genes

Plasmids isolated from the nine Bt native isolates by alkali lysis method [41] were utilized for the screening of *Cry*, *Cyt* and *PS* genes using specific primers (Table 3). The 25 µL PCR reaction mix comprised of 1 µL (30 pg) plasmid DNA, 2.5 µL 10× Taq buffer, 1 µL dNTP mix (10 mM), 1µL forward primer (10 pmol), 1 µL reverse primer (10 pmol), 2 µL Taq DNA polymerase (0.3 U/µL) and 17.5 µL sterile deionized water. Amplification was carried out in Applied Biosystems^®^ Veriti^®^ 96-Well Thermal Cycler with the following 30-cycle temperature program: initial denaturation, 94 °C for 5 min; denaturation, 94 °C for 1 min; annealing (Table 3) 55/65 °C 1 min; primer extension, 72 °C for 1 min; final extension, 72 °C for 10 min. PCR amplicons were resolved on 2% agarose, ethidium bromide-stained gel and subsequently visualized under the UV using by gel documentation system (UVITEC, Cambridge, UK). Plasmid DNA from *Bacillus thuringiensis* subsp. *israelensis* (*Bti-*H14) was included as a reference for comparison. 

### 4.11. Protein Chemistry

PS proteins were solubilized at a concentration of 0.2 mg/mL in 50 mM Na_2_CO_3_/HCl (pH 10.5) buffer containing 10 mM DTT. Protein concentration of the samples was measured using bovine serum albumin (BSA) as a standard [51]. Nascent parasporal pro-toxins were activated with trypsin at 1:10 enzyme to PS inclusion ratio (*v*/*v*) at 37 °C for 3 h and activated. PS crystal proteins were resolved on 12% Sodium dodecyl sulfate-polyacrylamide gel electrophoresis (SDS-PAGE) gel [52] and the bands were detected by silver staining according to standard procedure [53]. Molecular weights were determined by use of protein standard (Protein marker II, 6.5–200 kDa; Appli-Chem GmbH, Darmstadt, Germany) comprising myosin (200 kDa), β-galactosidase (116.3 kDa), phosphoryase B (97.4 kDa), bovine serum albumin (66.2 kDa), ovalbumin (45 kDa), and carbonic anhydrase (31 kDa) soybean trypsin inhibitor (21.5 kDa), lysozyme (14.3 kDa) and aprotinin (6.5 kDa).

### 4.12. Measurement of Cellular Viability by MTT Assay

The capability of the reducing enzymes that are present in viable cells to convert 3-[4,5-dimethylthiazol-2-yl]-2,5-diphenyltetrazoliumbromide (MTT) to formazan crystals was considered as a marker for measuring the cytotoxic potential of Bt-derived PS proteins against cancer cells according to previously described assay [54]. Briefly, cells cultured in complete medium were seeded into Thermo Scientific 96-Well Microtiter™ Microplates with 2 × 10^4^ cells per well and incubated at 37 °C under a humidified atmosphere of 5% CO_2_ for 24 h. The cell medium in test wells were then changed to serum free medium (SFM) containing 2-fold serially-diluted, solubilized and trypsinized, and Millipore^TM^-filtered (0.45 µm) PS proteins with concentrations of 25, 12.5, 6.25, 3.125, 1.56 and 0.78 µg/mL, while the cell medium in control wells were changed to SFM containing an equivalent volume of sodium carbonate buffer. After incubation at 37 °C under a humidified atmosphere of 5% CO_2_ for 24 h, cytopathic effects were observed under phase contrast microscopy. Then, SFM in control and test wells were replaced by 100 µL/well of MTT, 0.5 mg/mL) in Phosphate-Buffered saline (PBS) and incubated at 37 °C for further 3 h. Then, MTT was aspirated and the purple formazan crystals formed at the bottom of the wells were dissolved using 100 µL isopropyl alcohol/well with shaking for 1 h at room temperature in the dark. The absorbance at 549 nm was read on a microplate reader (ELX 800; Bio-Tek Instruments, Winooski, VT, USA). PS protein concentrations causing 50% cell growth inhibition (IC_50_) were calculated for each BT strain. The cytotoxic of the anticancer drug dasatinib, a potent multi-targeted kinase inhibitor of BCR-ABL and SRC family kinases [55], against the two cell lines was examined at the same concentrations of tested compounds and utilized as a standard positive control for comparative purposes.

### 4.13. Gene Expression Profiling of Apoptotic Genes by Quantitative Reverse Transcriptase Polymerase Chain Reaction (RT-qPCR)

Total RNA was extracted from HeLa cells, 24-h post-treatment with 12.5 µg of activated PS proteins isolated from Bt5, using Total RNA Purification Kit (Norgen Biotek Corp., Thorold, ON, Canada) according to the manufacturer’s instructions. RNA quality was assessed from the ratio λ_260_/λ_280_ by measuring the absorption at 260 nm and 280 nm (protein contamination) using Qubit® 2.0 Fluorometer and NanoDrop™ One^C^ Microvolume UV-Vis spectrophotometer (ThermoFisher Scientific™ GmbH, Karlsruhe, Germany); ratios were always >1.8. Genomic DNA was then eliminated and cDNA synthesized from RNA (1 µg) in a final reaction volume (20 µL) using the QuantiTect Reverse Transcriptase Kit (Qiagen, Germany). RT-qPCR was subsequently performed on a Rotor-Gene Q 5-Plex HRM thermal cycler (Qiagen, Germany) as previously described [40], using a QuantiTect SYBR-Green PCR Kit (Qiagen, Germany), with the following primer assays for apoptosis-related cysteine peptidase marker genes: *Hs_CASP3_1_SG QuantiTect Primer Assay* (QT00023947) and *Hs_CASP9_1_SG QuantiTect Primer Assay* (QT00036267) in a final reaction volume (25 µL) containing the diluted cDNA sample (5 µL), 2x SYRB-Green PCR Master Mix (12.5 µL), each forward and reverse primer (10 µM stock, 2.5 µL) and RNase-free water (2.5 µL). The amplification program and PCR amplicon specificity were performed and assessed as previously documented [40]. Each sample was represented by 2 biological replicas and three technical replicas, with the inclusion of a no-template control. Raw data were analyzed using the Rotor-Gene^®^ cycler software 2.1 (Qiagen GmbH, Düsseldorf, Germany) to calculate the threshold cycle (Ct) using the second derivative maximum. The fold-change value for each gene was determined after normalization to the expression levels of *18S* as a housekeeping gene which was calculated using the equation 2^−ΔΔ*C*t^ [56].

### 4.14. Statistical Analysis

The calculations and statistical analysis were carried out using the Statistical Package for Social Sciences (SPSS; IL, USA) for Windows version 17.0. All data were represented as mean ± standard deviation (SD) 3 individual experiments each in duplicate. The relative gene expression data were subjected to paired Student’s *t*-test in order to identify significant differences between PS- and Na_2_CO_3_-treated cells at equivalent dilution rate. Treatments were considered statistically significant at *p* < 0.05.

## Figures and Tables

**Table 1:** Biochemical typing of Bt isolates.

Isolate/Serial#	Geographical Location/Crystal Shape	SAC	AMY	VP	CIT	OX	MOT	ADH
**Bt1 (284)**	**Al Hasa**/Bi-pyramidal not attached	+	+	+	+	+	+	+
**Bt2 (319)**	**Hafr Al-Baten**/Attached hexagonal	−	+	+	+	+	−	+
**Bt3 (594)**	**Yonbaa**/Spherical	−	+	−	+	−	+	−
**Bt4 (605)**	**Al-Majma’ah**/Spherical	+	+	−	−	+	+	+
**Bt5 (M300)**	**Mecca**/Dark, bright, ovoid, Attached spore	+	+	+	+	+	+	+
**Bt6 (M268)**	**Al-Madina**/Hexagonal-spore in chain	−	−	+	−	+	−	−
**Bt7 (M13m)**	**Asir**/Small, outside, spherical, not attached	−	+	−	+	+	−	+
**Bt8 (M160)**	**Al-Majma’ah**/Large bi-pyramidal, attached spore	−	−	+	−	+	−	−
**Bt9 (224)**	**Al Taif**/Hexagonal, inclined spore	−	+	+	+	+	−	+

(SAC, sucrose; AMY, starch hydrolysis; VP, acetoin formation; CTA, citrate utilization; OX, oxidase; MOT, motility; ADH, arginine dihydrolase).

**Table 2:** Cytotoxic activity of different alkali-solubilized and trypsinized parasporal crystal proteins against cancerous cell lines 24-h post-treatment.

Bt isolate/Compound	HeLa		HT-29	
	Inhibition (%) *	IC_50_^#^ (µg/mL)	Inhibition * (%)	IC_50_ (µg/mL)
**Bt1**	38	3.2	3	nd ^a^
**Bt2**	40	5.7	2	nd
**Bt3**	35	5.4	0	nd
**Bt4**	38	5.4	2	nd
**Bt5**	40	9.5	3	nd
**Bt6**	42	14.2	0	nd
**Bt7**	47	5.6	0	nd
**Bt8**	40	6.4	0	nd
**Bt9**	43	6.1	4	nd
**Dasatinib**	58	24.4	nd	nd

Inhibition (%) *, Percentage of inhibition of cell proliferation at PS concentration of 12.5 µg/mL, relative to Na_2_CO_3_ buffer control at equivalent serially-diluted concentration; ^#^ IC_50_ values were calculated from the data of the cytotoxicity dose–response curve against HeLa cell line. nd ^a^, not determined. Dasatinib, is a positive control anticancer drug that acts as a potent multi-targeted kinase inhibitor of BCR-ABL and SRC family kinases.

**Table 3:** Characteristics of general and specific primers employed for the detection of *Cry*, *Cyt* and *PS* genes.

Primer	Sequence	Gene(s) Recognized	Amplicon Size (bP)	Condition of Annealing	GenBank Accession No	Reference
*Cry2* (UN)	(F)5′-GAGTTTAATCGACAAGTAGATAATTT-3′ (R)5′-GGAAAAGAGAATATAAAAATGGCCAG-3′	*Cry2Aa*	526	50 °C	M31738	[41]
		*Cry2Ab*	526		M23724	
		*Cry2Ac*	520		X57252	
		*Cry2Ad*	500		AF200816	
*Cry4* (UN)	(F)5′-GCATATGATGTAGCGAAACAAGCC-3′ (R)5′-GCGTGACATACCCATTTCCAGGTCC-3′	*Cry4A2*	439	58 °C	D00248	[27]
		*Cry4B4*	439		D00247	
*Cry4A* (spe)	(F)5′-TCAAAGATCATTTCAAAATTACATG-3′ (R)5′-CGGCTTGATCTATGTCATAATCTGT-3′	*Cry4Aa*	459	50 °C	Y00423	[27]
*Cry4B* (spe)	(F)5′-CGTTTCAAGACCTAATAATATAATACC-3′ (R)5′-CGGCTTGATCTATGTCATAATCTGT-3′	*Cry4Ba*	321	50 °C	‘X07423	[27]
*Cry10* (spe)	(F)5’-TCAATGCTCCATCCAATG-3′ (R)5’-CTTGTATAGGCCTTCCTCCG-3′	*Cry10*	348	51 °C	M12662	[27]
*Cry11* (UN)	(F)5’-CGCTTACAGGATGGATAGG-3′ (R)5′-GCTGAAACGGCACGAATATAATA-3′	*Cry11Aa*	342	50 °C	M31737	[41]
		*Cry11Ba*	342		X86902	
		*Cry11Bb*	452		AF017416	
*Cyt1* (UN)	(F)5′-CCTCAATCAACAGCAAGGGTTATT-3′ (R)5′-TGCAAACAGGACATTGTATGTGTAATT-3′	*Cyt1Aa*	477	52 °C	X03182	[41]
		*Cyt1Ab*	480		X98793	
		*Cyt1Ba*	477		U37196	
*Cyt2* (UN)	(F)5′-ATTACAAATTGCAAATGGTATTCC-3′ (R)5′-TTTCAACATCCACAGTAATTTCAAATGC-3′	*Cyt2Aa*	356	50 °C	Z14147	[41]
		*Cyt2Ba*	355		U52043	
		*Cyt2Bb*	355		U82519	
		*Cyt2Ca*	355		AAK50455	
*PS1Aa1*	(F)5′-TGTGCGATTGGTGGATGCGCT-3′ (R)5′-TCCCCGAAAAAGACCTGCGGT-3′	*Cry31Aa1*	226	65 °C	AB031065	[15]
*PS2Aa1*	(F)5′-TCCCAAAAGAGTAGGGCCAGGTG-3′ (R)3′-AATTCCCCCATTTTGGGCATTGGCA-3′	*Cry46Aa1*	269	63 °C	AB099515	[41]
*PS3Aa1*	(F)5′-GCCGGAATTGCTGGCCTCGA-3′ (R)5′-TGATGGGCTCCGTAGGTAGGGA-3′	*Cry41Aa1*	447	52 °C	AB116649	[4]
*PS4Aa1*	(F)5′-GAGGTGGTGTGCTGCAAGGGG-3′ (R)5′-TTCCCGAACCTGCCCTGCAC-3′	*Cry45Aa1*	712	57 °C	AB180980	[17]
